# Promoting functional foods as acceptable alternatives to doping: potential for information-based social marketing approach

**DOI:** 10.1186/1550-2783-7-37

**Published:** 2010-11-10

**Authors:** Ricky James, Declan P Naughton, Andrea Petróczi

**Affiliations:** 1 Kingston University, Faculty of Science, School of Life Sciences, Penrhyn Road, Kingston upon Thames, Surrey, KT1 2EE, UK; 2 The University of Sheffield, Department of Psychology, Western Bank, Sheffield, S10 2TN, UK

## Abstract

**Background:**

Substances with performance enhancing properties appear on a continuum, ranging from prohibited performance enhancing drugs (PED) through dietary supplements to functional foods (FF). Anti-doping messages designed to dissuade athletes from using PEDs have been typically based on moralising sport competition and/or employing scare campaigns with focus on the negative consequences. Campaigns offering comparable and acceptable alternatives are nonexistent, nor are athletes helped in finding these for themselves. It is timely that social marketing strategies for anti-doping prevention and intervention incorporate media messages that complement the existing approaches by promoting comparable and acceptable alternatives to doping. To facilitate this process, the aim of this study was to ascertain whether a single exposure knowledge-based information intervention led to increased knowledge and subsequently result in changes in beliefs and automatic associations regarding performance enhancements.

**Methods:**

In a repeated measure design, 115 male recreational gym users were recruited and provided with a brief information pamphlet on nitrite/nitrate and erythropoietin as a comparison. Measures of knowledge, beliefs and automatic associations were taken before and after the intervention with at least 24 hours between the two assessments. The psychological tests included explicit measures of beliefs and cognitive attitudes toward FF and PED using a self-reported questionnaire and computerised assessments of automatic associations using the modified and shortened version of the Implicit Association Test.

**Results:**

The information based intervention significantly increased knowledge (*p *< 0.001), changed explicit beliefs in specific FF (*p *< 0.001) and shifted the automatic association of FF with health to performance (*p *< 0.001). Explicitly expressed beliefs and automatic associations appear to be independent.

**Conclusion:**

Evidence was found that even a single exposure to a persuasive positive message can lead to belief change and can create new or alter existing associations - but only in the specific domain. Interventions to change outcome expectations in a positive way could be a rewarding avenue for anti-doping. Effective social marketing campaigns for drug free sport should follow appropriate market segmentation and use targeted messages via promoting the natural form as opposed to the purified form of the main active ingredient.

## Background

Following almost three decades of research, doping has now raised the attention of health professionals beyond the sporting arena, voicing concerns about doping use on the grounds of protecting physical and psychological well-being of athletes and non-athletes alike [1]. This view is mirrored in publications on doping in sport emphasizing the growing need for effective prevention [2], making a much needed shift from moral reasoning to general health concerns [3,4], or, at least, implementing harm reduction strategies [4-7] as realistic and sustainable solutions, with a strong focus on athletes' health [2].

The World Anti-Doping Agency (WADA) was established in 1999 to promote drug-free sport and to coordinate and monitor the fight against doping. To date, the prevailing approach to ensuring drug free sport is based on the three key documents (The World Anti-Doping Code, International Standards, and Models of Best Practice and Guidelines), each aiming to ensure harmonised detection and sanctions in nations that are signatories of the WADA anti-doping programme [8]. In recent years, this detection-based deterrence has been complemented with educational initiatives and social marketing campaigns. Despite the clearly stated organisational philosophy declaring that "a long-term solution to preventing doping is through effective values-based education programs that can foster anti-doping behaviours and create a strong anti-doping culture" [9], advances in this area are seriously lagging behind those made on the analytical side for drug testing. This discrepancy may partly arise from the magnitude of investment made into each area independently (approximately 3:100 benefitting 'science' over education and social science research together [10]), but also from the facts that i) the link between the goals and means of the education and awareness campaigns, by default, is less straightforward than it is for the analytical tests and ii) the desirable outcome (i.e. drug free sport) cannot be accurately ascertained.

Athletes are mainly thought to be vulnerable to doping in situations where much depends on sporting success [11]. However, the notion of assisted performance enhancement is not confined within the boundaries of highly competitive sport. As a direct result of this demand, the number of Internet retailers and range of products has mushroomed over the years and is now causing great concerns for safety [12-14]. Experimenting with various supplements is natural to most athletes as it is evidenced by the significant proportion of athletes reporting regular use; in many cases, polypharmacy [15-19]. The use of prohibited performance enhancements is an unwanted extension of this avenue [20-22] on which athletes have been progressing for quite a long time. It has been suggested that an effective and sustainable anti-doping approach may succeed if comparable acceptable means are offered along with the prohibition approach, intervening by changing outcome expectancies pertaining to doping and non-prohibited alternatives [21]. In this paper we take the first step in exploring the viability of this 'alternative means' approach.

When members of the exercise and athletic community decide which genre of supplements to use, they tend to make choices via said expected outcomes. If the outcome is perceived to be positive then it increases the likelihood of following with action whereas if the outcome is perceived as negative, the likelihood of making that choice is reduced. Therefore the process of choice involves weighing up positive outcome perceptions against negative ones. Positive and negative outcomes can be direct, for example physical enhancements or detrimental effects; as well as indirect outcomes such as fame and fortune or damnation.

Although social marketing, which uses commercial marketing techniques and strategies to influence people's behaviour for a greater public good, is still in its relative infancy, it has been effective across a wide range of public health areas including healthy lifestyle and health promotion, nutritional habits, obesity, drug use, smoking, alcohol consumption, road safety: speeding and risk/drink driving, condom use and HIV [23-34]. A fairly recent assessment of social marketing in anti-doping campaigns has reported the absence of social marketing but expressed a view in which social marketing would enhance the current detection-sanction as well as educational approaches to drug free sport [35]. This view is supported by a Europe-wide survey prepared for the European Commission on fighting doping [36] and a recent analysis of the anti-doping campaigns of UK Olympic Federations [37] indicating that whilst a considerable variation exists in anti-doping provisions, these campaigns tend to rely on information booklets, information service and workshops/seminars focusing on the moral aspect of doping with appropriate market segmentation and targeted messages mostly missing. Tailored and interactive campaigns designed and implemented by highly trained professionals have been recommended [38].

The ways in which social marketing strategies are best used in relation to doping are open to debate. Despite the use of secondary sourced information by various campaigns to deter athletes as well as the exercise population from using performance enhancing drugs (PED) [39], little is known about the most effective way to communicate messages that promote abstinence from PED use, whether it is for health, moral or legal reasons, although the latter one has been shown to have a lesser effect on athletes' decisions in hypothetical scenarios [40]. In the past anti-doping messages were typically produced in two forms: i) moralising sport competition or ii) employing scare campaigns, involving informing only the negative outcomes so that they outweigh the positives. The effectiveness of this approach depends on a plethora of external and internal factors, such as level of fear, framing, vivid presentation, physical versus social consequences, specificity, referencing, argument strength, source credibility, number of exposures, individual differences, emotions and goals [41]. With regard to PEDs, this approach has been shown not to yield any significant benefit in terms of deterrence whereas campaigns which provide secondary information in a more balanced manner have been shown to significantly increase agreement on adverse effects of PEDs [42]. These campaigns may help inform athletes of benefits and risks but fail to suggest acceptable alternatives.

Intervention strategies used in public health domains range from promoting positive examples to evoking fear, often using a combination of media. Reviews and meta-analyses [26,34,41,43-48] suggest that, among many other factors, the credibility of the source appears to be important for those that have no direct involvement in the target behaviour. Whilst there appears to be a consensus regarding the importance of 'framing', the type of framing that leads to the desired behaviour or behaviour change is much debated. It was noted that 'negative' messages are better recognised, regardless of the content or effect. Involvement and relevance certainly mediated the effectiveness, as well as the process between the type of message (e.g. gain or loss framing, fear arousal, comparative alternatives, perceived vulnerability, health, legal and social consequences) and outcome. Interestingly, some studies have found that fear appeal and negative perception of the message had reverse effects (hence were counterproductive) but this was not always the case.

In summary, in order to be effective, social marketing for anti-doping should use strategies developed and successfully employed in commercial marketing for decades, namely: deep understanding and consideration of information processing, inter-individual and developmental differences in decision making, appropriate segmentation for targeted messages. It is timely that anti-doping prevention and intervention incorporate media messages that, in addition to promoting drug-free sport for the sake of fairness or health, also propagate comparable and acceptable alternatives to doping. To facilitate this process, we test the effectiveness of a knowledge-based information intervention in changing beliefs regarding performance enhancements.

## Methods

The experimental procedure was approved by Kingston University Faculty of Science Research Ethics Committee. The participation was voluntary with anonymity assured after data collection by coding the responses and removing all identifiable personal information.

All participants were fully informed of the potential benefits, risks and time requirements. Once all documentation had been received and read, an informed consent form was signed.

The psychological tests included explicit measures of beliefs and cognitive attitudes toward functional foods (FF) and PED using a self-reported questionnaire and computerised assessments of parallel implicit cognitions using the modified and shortened version of the Implicit Association Test (IAT) [49,50].

### Information leaflet

The information leaflet provided fact-based information on nitrate and erythropoietin as a comparison. (Additional file 1: Information pamphlet provided to participants on physiological effect or nitrate-rich food [beetroot] and a comparable 'synthetic' drug [erythropoietin]).

### Questionnaire

The questionnaire consisted of five main sections. The first section contained a variety of functional foods and chemical based supplements (obtained from a word association task), volunteers were asked to tick if they believed they were good for strength, endurance, both, useless or don't know. The second section, where questions were specific to nitrate supplementation (administration, side effects, etc), was assessed on the number of correct answers. The third section focused on information sources, where participants had to select where they sourced their information about supplementation. In the fourth section, participants were required to rate how much they believed a FF or PED would work from the same category, for example guarana and 'speed' are both with stimulating effect. Gym users were required to answer on a 7-point Likert-type scale on how stimulating they think these substances were individually. The categories were stimulation, endurance, strength, overall competitiveness and overall performance (5-point scale). The focus was on endurance, competitiveness and overall performance but the other two were added to ascertain if a change would occur in belief about FF and other performance attributes. The fifth and final section required subjects to put examples of fruit and FF found on the pamphlet, into categories of health or functionality.

### Brief implicit association test

Association tests require people to sort words to pre-identified categories as accurately and fast as possible. Participants are not required to make any connection between the words and attributes, only to categorise each correctly within its own domain (i.e. target words into categories as PED or FF and attributes into categories such as 'healthy' or 'performance enhancing'). The IAT concept has been used to detect food preferences [51] and variations of the implicit association test have been adapted to doping [52] and used in doping research [53-55].

In this project, a modified Brief IAT was used [50] using word stimuli. This is the first application of the implicit cognition measures pertaining performance enhancing substances (PED and FF) that diverge from the classic good/bad or pleasant/unpleasant associations and taps into cognitive attitudes by using associations between different categories of performance enhancing substances (PED and FF) and performance enhancing/healthy attributes. The implicit association test (abbreviated as FF - H/P) was used to ascertain if recreational gym users would associate functional foods with performance or health; and whether this changed after the information intervention. In this test, the two target categories were Fruits (*Apple, Orange, Kiwi, Banana*) and Functional Foods (*Celery, Spinach, Lettuce, Beetroot*), with Fruits being non-focal. Attributes were Healthy (*Vitality, Healthy, Vigour, Wellbeing*) and Performance (*Speed, Strength, Endurance, Flexibility*). Participants were instructed to categorise defined combinations of the focused target and attributes (giving *Functional food + Healthy *and *Functional food + Performance *pairings) by pushing a dedicated key on the keyboard whilst pushing an alternative key for 'everything else'. The non-focal target category, serving as a balance in the 2 × 2 design, only appears in the 'everything else' instruction [50] and thus it does not contribute to the implicit association measure. The latency measures were converted into D scores with the following interpretation: Functional foods - Health (indicated by a negative number) or Performance (indicated by a positive number).

The strength and direction of the association between the target words and attributes is shown by D scores, which ranges between +1 and -1. A positive number indicates that the subject has a strong association with target A with attribute A or target B with attribute B, a negative number indicates that the subject has a strong association with target A with attribute B or target B with attribute A. The closer the D score is to +1 or -1 indicates the strength of this association [50,56]. The advantage of the D score is that it affords protection against the general cognitive ability confound [57]. The interpretation of the D score is in line with Cohen's conventional effect sizes of small (d = 0.2 - 0.3), medium (d = 0.5) and strong (d > 0.8) effects [58].

### Participants

Volunteers were recruited among body builders, athletes and recreational gym users. Specific inclusion criteria were that subjects were male (to avoid inter-group differences by gender), and had some knowledge of and/or experience with supplementation. The first part of the study involved 236 males recruited for a word association task (data not shown). Results from this phase were used to inform the FF - H/P and questionnaire. Participants in this part of the study were between 18 to 38 years of age. The second part of the study involved 115 male recreational gym users recruited independently from the first study, who were recruited to ascertain if information can affect attitudes towards functional foods as well as increase an individual's ability to differentiate between healthy foods and functional foods. Participants in this part of the study ranged from 18 to 45 years of age. Participants in both studies were asked if they had experience and/or general knowledge of nutritional supplements and those with affirmative answers were included in the sample. This knowledge was not formally assessed.

### Study design

In order to gain insight into the most widely known performance enhancing supplements and healthy foods, male patrons of a local gymnasium were asked to give 5 examples in each category: healthy foods, muscle building and endurance supplementation. The most frequently occurring supplements and foodstuffs were used in the construction of the FF - H/P and the questionnaire.

Following the first phase, healthy male participants were recruited to take part in the experimental phase. This part of the study required participants to complete a self-report questionnaire and the computerised brief implicit assessment task twice. The first pre-intervention FF - H/P and questionnaire were measured to get a baseline. Subjects were then given an information pamphlet on nitrate supplementation as part of the Participant Information of the experimental study. Participants were asked to take the information home and return the following day (or few days) if they wished to participate. Upon return, participants were asked to complete the same questionnaire and implicit test. At least 24 hours elapsed between the two tests, allowing participants to read and absorb the information.

The Information Sheet explained that at a later stage, volunteers will be required for a nitrate study involving supplementation and two 10 mile (16 k) cycling time trials (data not shown). This combined approach afforded presenting the information on nitrate/nitrite and erythropoietin (used for comparison of physiological effects) as part of the Participant Information pack; hence participants were unaware that the information leaflet itself was part of the experiment.

### Statistical analysis

Reaction times on the FF - H/P tasks were recorded. Strength and direction of implicit association were shown using D-scores [56,59] calculated as the difference in mean response times divided by the variance of all measured latency. Paired samples t-test and nonparametric test (Wilcoxon Signed Rank) were used to analyse differences between the pre- and post-intervention measures. Owing to the nature of measurement used in some variables, nonparametric correlation coefficients (Kendall tau) were used to test for relationships between the change in knowledge and attitude measures. The overall α level was set at 0.05.

### Equipment

The FF - H/P task was run on a Samsung R530 laptop using Inquisit software version 3.0.4.0 (Milliseconds) under Windows XP operating system. Response options were assigned to keyboard letters. The questionnaire was designed and hosted on a surveymonkey professional account. All statistical analyses were performed using PASW Statistics 17.

## Results

The mean age in the information intervention study was 23.35 (SD = 5.445). Participants were mainly recreational gym users (108/115) attending the local health club regularly.

### Information source

Based on the answers provided by the recreational gym users in this study, the Internet (54/115) appears to be the dominant source of information on potential performance aids, followed by training partners (47/115) and friends (44/115). The numbers of selections in these three top categories were identical in the baseline- and follow-up questionnaires. Coaches, family, fitness and/or specific sport magazines, television and information pamphlets appear to be insignificant sources of information with less than 3% of participants selecting any of these sources. Interestingly, the information pamphlet as source of information was selected by 3 respondents for the post intervention, in comparison to none at the baseline measure.

### Knowledge

Post information-intervention knowledge was shown to increase in three key areas. Correctly answered questions on nitrate supplementation showed a significant increase (Z = -8.397, *p *< 0.001) with 77% achieving a higher score on the post information-intervention test. The remaining 23% did not show improvement but nobody performed worse on the second test (1 answer missing). In addition, the number of correct answers in recognising foodstuffs as functional foods significantly increased (Z = -9.012, p < 0.001) but apparently this happened at the expense of the foodstuff being concurrently recognised as 'health oriented' (Z = -0.250, p = 0.803) in some 40% of the cases. More specifically, whilst great improvement was shown in 93% percent (106 improvement, 7 ties, 1 decrease, 1 missing) correctly classifying a foodstuff as functional food, there was a considerable change in classifying the same as health *and *function oriented: 43 respondents changed from 'both' to the functional oriented only option, 42 did the opposite with 29 ties and 1 missing. These results suggest that either the 'both' option was used when respondents were uncertain or people may prefer 'clean' categories as opposed to holding a foodstuff in two equally valid mental categories. Answers given to the question on the specific function of nitric oxide: whether it is to increase strength, endurance, both or being useless, showed that 74% (n = 84) of the respondents have learned something about erythropoietin (which was only used for comparison) in contrast to the intended increase in knowledge for nitric oxide, where learning was only evidenced in 3 (2.6%) cases. Apparently, as an unintended consequence, the pre-existing difference in knowledge regarding EPO and nitric oxide (correct answers logged as 17 vs. 5, respectively) was magnified by providing information on both, despite the health option focus of the information material.

### Beliefs and attitudes

Results from the questionnaire showed explicitly declared beliefs and attitudes of the recreational gym users in the sample. The majority of the respondents believed that those on the WADA List of Prohibited Substances are effective for performance enhancement (extremely effective: 17.4%, fairly effective: 21.7%, effective: 26.1%, somewhat effective: 29.6%, not at all effective: 5.2%) and this view did not change after the information intervention. At the baseline measure, a considerable proportion of the respondents (73/115) felt that functional foods are not comparable healthy alternatives to doping. After the information intervention, 37 of these have changed their view resulting in a reversed balance between those who believed in FF as comparable alternatives to doping (78/114) and those who do not.

**Figure 1:**
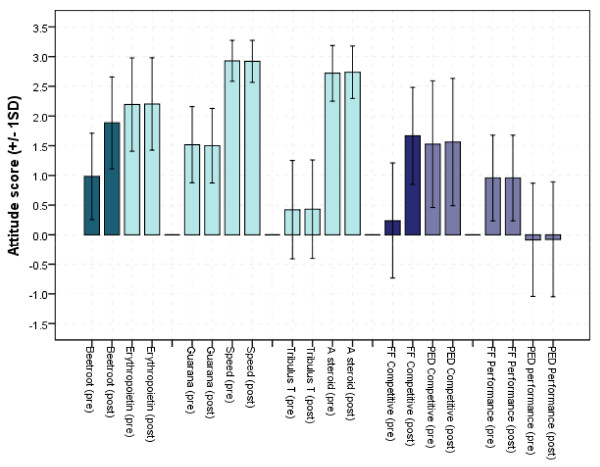
**Average explicit attitude scores before and after the information intervention**. Green: performance specific substances; purple: general questions; dark columns show where change occurred.

Two belief measures were shown to increase (Figure 1). Belief in beetroot juice as an endurance performance aid significantly increased (Z = -6.312, *p *< 0.001) as well as belief in functional foods as an overall performance enhancer (Z = -7.601, p < 0.001). Overall 51 and 75 respondents increased their ratings respectively after the intervention with 36 and 63 ties. Reversed effect (lower ranking after intervention only occurred in 3 cases, limited to the general question of FF increasing competitiveness).

**Figure 2:**
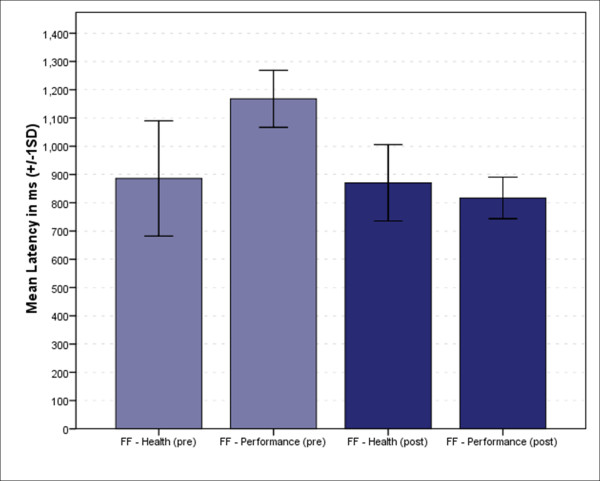
**Average latency in milliseconds measured on performing the FF - H/P test before and after the information intervention**.

**Figure 3:**
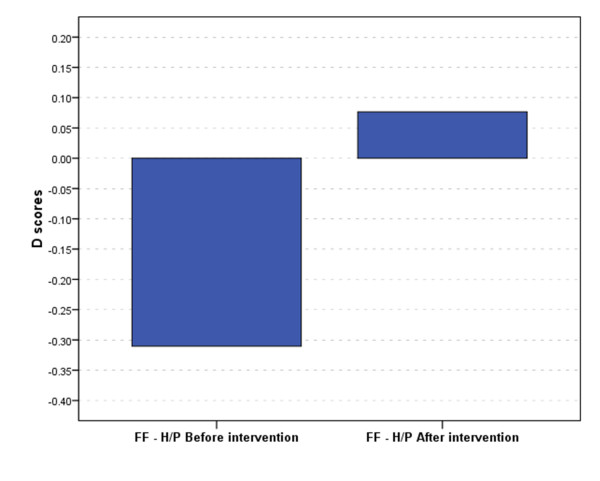
**D scores of the FF - H/P test before and after the information intervention**.

Implicit association was based on response latency measures on the FF - H/P tasks where functional food was paired with health and performance. Figure 2 depicts the average latency in each pairs in the FF - H/P task, before and after the intervention, whereas Figure 3 shows the corresponding D scores. Analysis of the pre-intervention data showed a greater preference for health in relation to functional food (Mean = 885.87 ± 203.88 ms in comparison to Mean = 1167 ± 100.89 ms averaged on the functional food - performance pair). This preference disappeared or even slightly reversed (Mean = 870.49 ± 135.15 ms vs. Mean = 817.08 ± 73.61 ms), after the information intervention focusing on performance enhancing properties of the selected functional foods. Figure 2 also shows that respondents performed the FF - Health pair with similar average time (885.87 ± 203.88 ms and 870.49 ± 135.15 ms for pre-and post intervention, respectively, t = 0.689, p = 0.492) but with a significant reduction in response time in the FF-Performance pair (1167.79 ± 100.89 and 817.08 ± 73.611 for pre-and post intervention, respectively, t = 29.604, p < 0.001).

Comparing the D-scores (Figure 3) which take cognitive ability into account, the difference between pre- and post intervention measures for FF being functional vs. healthy food (t = -17.578, *p *< 0.001) was statistically significant. Pre-information intervention, subjects exhibited medium associations (D = -0.310) between functional foods and health, which has changed to weak associations with performance (D = 0.077) after the information was provided on beetroot. Correlations between explicit and implicit measures; and between knowledge and attitude measures, were small and not significant.

Beliefs regarding and implicit associations toward functional food appear to be malleable in the short term. Changes in favour of seeing functional food as a potential performance enhancer (as opposed to a healthy option) were observed in both explicit and implicit measures after the intervention. This is somewhat contrary to the expected effect based on literature precedence [60] but consistent with the increased knowledge regarding functional food and specifically, nitrate rich foodstuffs and their physiological and performance enhancing effect. It is notable that changes in explicitly expressed beliefs regarding specific substances only occurred in one of the three: beetroot which was used in the information pamphlet. This effect has generalised to competitiveness but not to performance.

## Discussion

This study suggests that the type of information provided along with the timeframe was sufficient enough to increase knowledge on nitrate supplementation and on EPO which is a prohibited substance with similar performance enhancing effect. The fact that there was also an (unplanned) change in knowledge pertaining EPO could be due to the direct comparison used in the pamphlet. Providing comparisons can allow subjects to gauge how effective a supplement could potentially be. However, this approach appeared to be a double edged sword as on one hand, as it allowed FF to have a PED comparison to also focus on, it may increase the perception of it as a valid alternative but on the other hand, it might alert people to a potential drug.

The information provided was enough to change beliefs towards beetroot supplementation but not the other healthy alternatives; again this could be because of the direct comparison to EPO as well as the fact that beetroot (the example used in the information pamphlet) is not a very common everyday vegetable. As previously stated it is hard for consumers to believe that everyday type products can be used as performance aids just by changing 'dosage' and administration. The information included research concerning nitrate in beetroot juice but the question remains whether this information automatically translates to all nitrate rich foodstuffs. Further studies, using different foodstuffs such as salads, spinach or tomatoes, are required to gain a better insight into this effect.

The results provided evidence that knowledge (achieved via a meaningful message), in fact, is linked to beliefs and implicit attitude formation. In the Theory of Planned Behaviour framework [61], attitude is defined as a decisional balance between pros and cons about performance enhancing substances. Attitudes, complemented by subjective norms and perceived behavioral control, lead to behavioural intentions and progress to volitional phase, if the situation for the act is favorable. Perceived behavioural control is equivalent to the combination of outcome expectancies and construct specific self-efficacy [62], such as doing well without the assistance of performance enhancing substances. In other words, whilst self-efficacy is a belief in self to successfully execute the behavior required for the desirable outcome, outcome expectancy refers to one's estimation that this behavior will, indeed, lead to the desired outcomes. Therefore, athletes who wish to use performance enhancing substances but prefer to refrain from the prohibited ones must believe that i) they are able to remain competitive without prohibited substances and ii) alternatives (dietary supplements and functional foods) are, indeed, comparable alternatives. Congruently, those who contemplate using or use PEDs must believe that these alternatives are inferior to the prohibited substances and that they would not remain competitive if doping is not used. Assuming that the message is moderated via personal preferences and experiences, affording greater influence on some more than others, in addition to the characteristics of the 'message' (information), it is assumed that athletes' attitudes, outcome expectancies (beliefs about PEDs and FF), motivation toward the importance of performance enhancements within or beyond the permitted means, and their self-efficacy, may serve as moderators in information processing.

The results also indicate that individuals prefer to gain their information from peers and websites. This can prove problematic if the person they gain their information from is already affiliated with PED's. As PEDs are not available from shops and blindly asking the wrong person may result in disapproving looks. For example, access to anabolic steroids has been shown to act as a barrier to use [63]. In order to gain access to PEDs, individuals are likely to have some association with individuals who are able to gain access. These key information sources should be taken into consideration in targeted social marketing campaigns. Messages using the Internet must be produced in a way that fits to the interests of those who wish to find information about alternatives to PEDs. Social marketing tools may also incorporate means that encourage an online community of alternative performance enhancement users to grow. This will increase the likelihood of information being passed on via word of mouth.

The importance of fact-based, accurate information is underscored by results from recent investigations that highlighted the considerable mismatches that exist between choices of nutritional supplement and reasons for their use among diverse high-performing athletic populations [64-66]. Given the importance of nutrition and the expert support available for these populations, the lack of rationale behind their choices of supplementation is alarming. This position suggests that athletes' perceptions of dietary supplements with performance-enhancing properties may be made on questionable grounds such as limited and overemphasized information in the media and highlights the scale of piecemeal guidance, often dubious or incorrect, that is readily accessible by the user. This scenario may also be interpreted as a discrepancy between athletes' choices, industry information, marketing and academic specialists regarding ergogenic aids. Whilst the multilevel causes of this disagreement involve a number of known parameters such as accuracy of marketing information, accessibility of scientific information, opinion leadership, price or availability, one additional key determinant may be the moderating factor that influences the information process on the receiver's end.

The somewhat surprising result regarding the change in both explicitly expressed beliefs and automatic associations might be explained by the potentially magnified interest. Previously, new automatic association has been found after a single exposure to a short written story [67] suggesting that a persuasive message leading to newly acquired knowledge can create new or alter existing associations. Although not directly tested in this study, it is also plausible that the context in which the information was presented (i.e. recruitment for an exercise physiology trial testing the effectiveness of nitrate rich functional food on endurance), this new knowledge structure may also initiate implementation intentions, which have been shown to effect could promote control over implicit associations [68].

Regarding limitations, for practical reasons the study was conducted among users of a university gym in a large city. All participants were male within an academic community with associated levels of education. It also should be noted that the researcher collecting the data, although not friends with any of the subjects has had occasional contact with them and could be perceived as someone who knows about supplementation. Yet this further supports community based information. It can also be argued that the dimension of evaluation (healthy vs. performance enhancing) is favouring functional foods. However, exercise physiology literature is brimming with experimental studies using foodstuff, fruits and vegetables alike, to find natural sources of performance enhancing substances. For example, red berries are generally known for their antioxidant properties with recent studies looking into tart cherries to prevent symptoms of muscle damage [69].

Future directions arising from this study relate to testing the effect of direct experience on implicit and explicit attitudes, as well as investigating the stability of the observed change over time. The current study does not offer insight into behavioural intention or volition. Follow up studies should elucidate how attitude change upon vicarious or direct positive experience with functional food lead to behaviour change; and whether it will happen is a desirable direction.

## Conclusion

Effective PED deterrence campaigns should accept that a desire for constant performance enhancement is natural to athletes. Instead of a solely prohibitive approach, anti-doping campaigns should promote acceptable and healthy alternatives to doping and primarily seek to create a community that takes the Olympic spirit further.

Promoting the natural form (as opposed to the purified form of the main active ingredient) is key to the 'alternative means' approach. In the unrelenting quest for effective but not prohibited substances, athletes may put their health in great danger. There is a wide range of risks associated with the use of performance enhancing substances that do not apply to naturally occurring functional foods which mainly arise from the omission of the concentration step converting the foodstuff to a supplement or allegedly pure therapeutic agent with dosage ramifications. Improvements in our understanding of nutrigenomics and pharmacogenomics warrant caution regarding use of concentrated substances in supplement form. Owing to variations in genetic make-up the effect of a quantity of a supplement can vary enormously in pharmacodynamic and pharmacokinetic effects leading to large variations in therapeutic efficacy along with toxicity profiles.

One of the criteria for a drug to be included into the list of prohibited substances is that it presents a danger to health. Functional foods, whilst aiding athletic performance, are the opposite: they are healthy. The campaign should include an online community that can offer information about comparable healthy alternatives and spread this approach for benefits to all stakeholders. Also better information should be made available about FFs regarding dosage and administration. As FFs are becoming increasingly available in a variety of products [70], wide dissemination of accurate information would facilitate safe intake and thus prevent overdosing.

## List of abbreviations used

EPO: Erythropoietin; FF: Functional foods; FF H/P: Brief Implicit Association Test for Functional Food - Healthy/Performance; PED: Prohibited performance enhancing drugs.

## Competing interests

The authors declare that they have no competing interests.

## Authors' contributions

RJ was the primary investigator and was responsible for recruitment, data collection and statistical analysis, contributed to drafting the manuscript. AP initiated the study, contributed to devising the tests, interpretation of the results and drafted the manuscript. DPN contributed to the study design, devising the information leaflet on nitrate and drafted the section on functional food. AP and DPN supervised the study. All authors read and approved the final manuscript.

## Supplementary Material

Additional file 1 **Nitrate Information pamphlet**. Information pamphlet provided to participants on physiological effect or nitrate-rich food [beetroot] and a comparable synthetic drug [erythropoietin] Click here for file
