# Supplementary material for: Promoting functional foods as acceptable alternatives to doping: potential for information-based social marketing approach
Source: J Int Soc Sports Nutr. 2010 Nov 10;7:37. doi: 10.1186/1550-2783-7-37 (PMC2994790; doi:10.1186/1550-2783-7-37)

Natural sources of nitrate

There are various source of nitrate. The sources with the highest levels are root vegetables.

These include:

- Lettuce
- Carrots
- Celery
- Beetroot
- Radishes

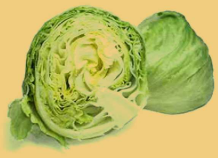

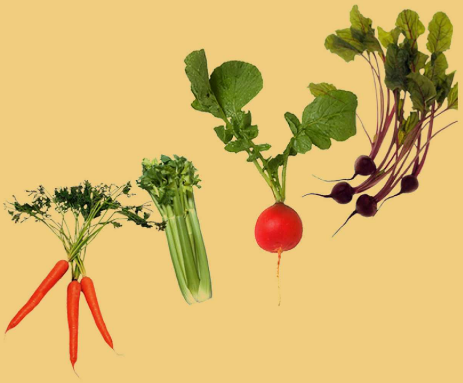

Side Effects

There are no documented side effects with the use of nitrate in a sport setting. Yet patients who have used nitrate in clinical practice have experienced the following:

- Headache,
- Dizziness,
- Flushing,
- Rapid heartbeat,
- Restlessness.

NITRATE

Information pamphlet

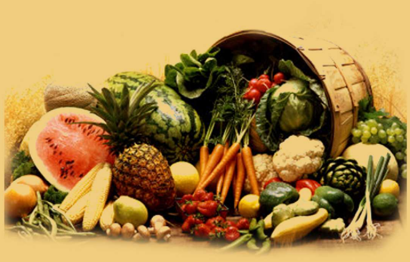

What is Nitrate & how does it work?

Nitrate can be found in various sources. Edible sources are mainly root vegetables. When a nitrate containing food is consumed the nitrate is converted into nitrite via bacteria in the mouth.

Nitrite is then swallowed and converted into nitric oxide. Nitric oxide relaxes blood vessels thus reducing blood pressure. Yet it is still unknown how it directly aids endurance.

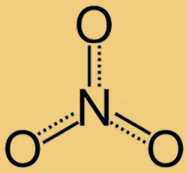

What are the benefits?

As this is a fairly new area there are only a few tested benefits of increasing dietary nitrate although research is ongoing.

It was found to decrease blood pressure.

It has also been found to reduce the amount of oxygen needed to perform the same activity.

It has also been shown to increase cycling until failure times. Some might say the time until failure results could be comparabile to results shown when using a prohibited drug called Erythropoietin.

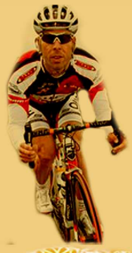

How should I take it?

There have been various studies that have used nitrate, the lowest levels used which yielded results was:

5.5 mmol (~340 mg) of nitrate per day

In the form of

500ml per day of beetroot juice.

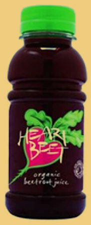

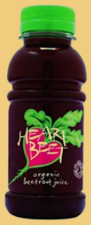

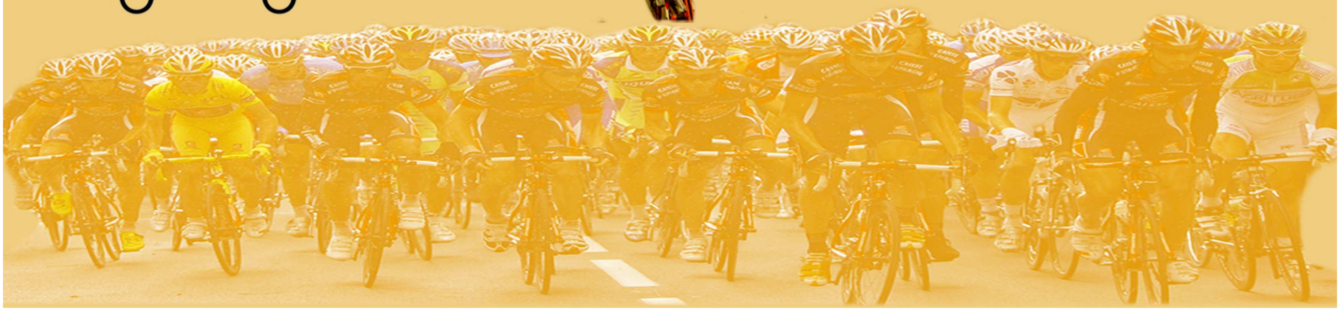

Supplement: Additional file 1 — Nitrate Information pamphlet. Information pamphlet provided to participants on physiological effect or nitrate-rich food [beetroot] and a comparable synthetic drug [erythropoietin] [file 1550-2783-7-37-S1.PDF]
